# Commentary: Static and temporal dynamic changes in brain activity in patients with post-stroke balance dysfunction: a pilot resting state fMRI

**DOI:** 10.3389/fnins.2025.1607090

**Published:** 2025-05-30

**Authors:** Xiaopeng Wu, Yanping Miao

**Affiliations:** Department of Radiology, The Affiliated Hospital of Inner Mongolia Medical University, Hohhot, China

**Keywords:** fMRI, post-stroke balance evaluation techniques, stroke, brain activity, resting state

Tang et al.'s (2025) recent investigation, “Static and temporal dynamic changes in brain activity in patients with post-stroke balance dysfunction: a pilot resting-state fMRI study,” constitutes a pioneering attempt to synthesize static and dynamic resting-state functional MRI (rs-fMRI) metrics with machine learning frameworks for dissecting the neural underpinnings of post-stroke balance impairments. While this study offers preliminary insights into the neurofunctional correlates of balance deficits, several methodological and interpretive shortcomings demand rigorous examination, particularly in light of the urgent clinical need for robust, actionable biomarkers in this domain.

## Key contributions and methodological gaps

The study's primary strength resides in its multidimensional analytical approach, examining six distinct rs-fMRI indices—including amplitude of low-frequency fluctuations (ALFF), fractional ALFF (fALFF), regional homogeneity (ReHo), degree centrality (DC), global brain connectivity (GBC), and dynamic functional connectivity (dFC)—across a cohort of 26 stroke patients and 24 age-matched healthy controls. This comprehensive metric selection permits a granular interrogation of both local and global neural dynamics, a strategy that aligns with contemporary models of postural control emphasizing distributed network interactions (Fox and Raichle, 2007). The observation of hypoactivity within visual-parietal and sensorimotor cortices, concomitant with compensatory hyperactivity in subcortical structures (notably the thalamus and basal ganglia), resonates with established theories of post-stroke neuroplasticity, wherein secondary brain regions may assume functional roles traditionally subserved by damaged primary areas.

However, the pilot nature of the study (total *N* = 50) introduces substantial statistical limitations. The reported correlation between lingual gyrus sALFF and Berg Balance Scale (BBS) scores (*r* = 0.41, *p* = 0.04) becomes non-significant upon false discovery rate (FDR) correction, underscoring the heightened risk of Type II errors in underpowered analyses (Button et al., 2013). To achieve sufficient power (≥80%) for detecting moderate effect sizes (Cohen's *d* = 0.5), future studies should recruit a minimum of 120 participants (60 per group), with balanced representation of stroke subtypes and comorbidities. Such preliminary findings, while hypothesis-generating, require validation in larger, demographically and clinically diverse cohorts to establish their clinical relevance. Furthermore, the absence of multiple comparison correction across all six rs-fMRI metrics may inflate the likelihood of spurious associations, a critical consideration given the exploratory nature of the study.

## Dynamic metrics: promise and pitfalls

The integration of dynamic analyses represents a notable methodological advancement, revealing transient compensatory mechanisms that static metrics may obscure. For instance, the identification of time-varying connectivity patterns between the default mode network (DMN) and salience network (SN) during resting-state suggests a dynamic reconfiguration of large-scale brain networks in response to balance challenges. However, the study's reliance on a conventional sliding window approach (window length = 50 TRs) is suboptimal. Emerging evidence from dynamic functional connectivity studies indicates that shorter windows (20–30 TRs) more effectively capture rapid neural fluctuations, particularly in clinical populations with altered temporal dynamics post-stroke (Lurie et al., 2020). Additionally, the absence of multi-window or adaptive parameterization strategies limits the interpretability and generalizability of the dynamic findings. Multi-window approaches (e.g., combining 20 TR, 30 TR, and 40 TR analyses) and adaptive parameterization (e.g., time-varying window lengths selected via spectral density analysis) could enhance sensitivity to context-dependent brain state transitions (Lurie et al., 2020). Adaptive parameterization strategies, such as time-varying window lengths selected via spectral density analysis, could further optimize temporal resolution while maintaining signal stability.

## Exclusion of critical neuroanatomical substrates

A glaring methodological limitation lies in the exclusion of patients with cerebellar and brainstem strokes. These regions are indispensable for sensorimotor integration, proprioception, and vestibular processing—cornerstones of balance control. While the study's focus on cortical/thalamic networks aligns with its hypothesis-driven aims, the exclusion of cerebellar/brainstem strokes introduces selection bias, potentially overemphasizing cortical contributions to balance dysfunction.

## Clinical translation and longitudinal insights

The cross-sectional design of the study precludes examination of neural recovery trajectories, a critical gap given the dynamic nature of post-stroke neuroplasticity. Balance impairments often evolve over months to years, with distinct temporal windows for spontaneous recovery and therapeutic intervention. Longitudinal rs-fMRI assessments, ideally paired with biomechanical measures (e.g., postural sway analysis) and clinical outcome scales, are essential to identify time-sensitive biomarkers predictive of long-term prognosis. Such a longitudinal approach could also elucidate the adaptive or maladaptive nature of observed neural changes, informing the development of targeted rehabilitation strategies.

## Machine learning and clinical integration

While the XGBoost model demonstrates diagnostic potential, interpretability remains a critical limitation. Feature importance rankings (e.g., SHAP values) could identify biomarkers with mechanistic relevance, balancing predictive performance with neurobiological plausibility. Hybrid models incorporating dynamic rs-fMRI indices, clinical variables (e.g., stroke severity, lesion volume), and biomechanical data may enhance diagnostic precision and prognostic utility. Moreover, the study's failure to validate findings in multi-center cohorts limits external validity, a critical consideration for clinical translation (Poldrack et al., 2017). Future work should prioritize multi-site validation to ensure generalizability across diverse patient populations and healthcare settings.

## Future directions

To advance the clinical translation of rs-fMRI biomarkers for post-stroke balance dysfunction, future research must address methodological limitations while embracing technological innovation. First, expanding cohort diversity through multi-center recruitment and explicit inclusion of cerebellar/brainstem stroke patients will mitigate selection bias and elucidate the contributions of subcortical neurocircuitry to balance control. This expansion should prioritize demographic heterogeneity (age, sex, comorbidities) and clinical variability (stroke etiology, lesion volume) to enhance generalizability. Concurrently, dynamic rs-fMRI analyses require refinement through shorter sliding windows (20–30 TRs) and adaptive parameterization strategies (e.g., spectral density-driven window selection), which have demonstrated superior sensitivity to rapid neuroplasticity mechanisms in post-stroke populations compared to conventional 50 TR approaches.

Complementing these methodological advancements, functional near-infrared spectroscopy (fNIRS) emerges as a critical tool for validating neuroimaging findings during ecologically valid balance tasks. Its motion tolerance and capacity for simultaneous behavioral monitoring make it particularly advantageous for studying cerebellar/brainstem stroke patients, who are prone to fMRI signal dropout in these regions. For instance, fNIRS could corroborate thalamic hyperconnectivity patterns observed in rs-fMRI while patients perform dynamic postural challenges, thereby bridging gaps between resting-state metrics and real-world functional outcomes.

Longitudinal study designs will further strengthen this framework by mapping temporal trajectories of neural recovery. Serial rs-fMRI assessments paired with biomechanical metrics (e.g., postural sway velocity) and clinical endpoints (e.g., Berg Balance Scale scores) could identify intervention-sensitive biomarkers, distinguishing adaptive plasticity from maladaptive compensation. Hybrid machine learning models integrating dynamic rs-fMRI features, lesion characteristics, and kinematic data would enhance diagnostic/prognostic precision, particularly when validated across diverse populations using rigorous statistical correction (e.g., FDR) to mitigate Type II error risks.

Ultimately, multimodal integration of fNIRS and fMRI biomarkers, coupled with advanced analytic pipelines, promises to transform pilot insights into actionable tools for personalized rehabilitation (Yang and Wang, 2025). This convergence of methodological rigor and technological innovation aligns with the NIH's vision for inclusive, clinically impactful neuroimaging research.

## Conclusion

Tang et al.'s study provides a foundational framework, but methodological refinements (e.g., larger cohorts, multi-window dynamic analyses, and cerebellar/brainstem inclusion) are essential to bridge the gap between neuroimaging discoveries and personalized rehabilitation strategies.
